# To Resurface or Not to Resurface the Patella in Total Knee Arthroplasty, That Is the Question: A Meta-Analysis of Randomized Controlled Trials

**DOI:** 10.3390/medicina58020227

**Published:** 2022-02-02

**Authors:** Alberto Delgado-González, Juan José Morales-Viaji, Jose Gregorio Arteaga-Hernández, Ángela Larrosa-Arranz, Guillerno Criado-Albillos, Adoración del Pilar Martin-Rodríguez, Maha Jahouh, Josefa González-Santos, Leticia Mendieta Díaz, Carla Collazo Riobo, Sara Calvo Simal, Jerónimo Javier González-Bernal

**Affiliations:** 1 Traumatology and Orthopedic Surgery Department, Burgos University Hospital, 09006 Burgos, Spain; alberto.delgado.med@gmail.com (A.D.-G.); jjmoralesviaji@gmail.com (J.J.M.-V.); jgarteagah@hotmail.com (J.G.A.-H.); alarrosa01@gmail.com (Á.L.-A.); guillecrial@hotmail.com (G.C.-A.); amartinro@saludcastillayleon.es (A.d.P.M.-R.); letimendiaz@gmail.com (L.M.D.).; carlacollazoriobo@gmail.com (C.C.R.); scalvo@hubu.es (S.C.S.); 2 Department of Health Sciences, University of Burgos, 09001 Burgos, Spain; jejavier@ubu.es

**Keywords:** patella, knee, resurface, arthroplasty

## Abstract

*Background and Objetives*: Currently, total knee arthroplasty is one of the most common surgeries, increasing with the increase in life expectancy. Whether or not to replace the patella has been a subject of debate over the years, remaining in controversy and without reaching a consensus. Over the years, different meta-analyses have been carried out in order to provide evidence on the subject, although, in recent times, there have not been many new studies in this regard. Therefore, it is considered necessary that the latest works form part of a new meta-analysis. *Materials and Method*: We searched the literature using PUBMED, SCOPUS, the Cochrane database and VHL from 2010 to 2020. The search terms used were “patellar” AND “resurfacing” OR “Replacement” and “no resurfacing” OR “no replacement”. A meta-analysis was performed with Stata software (Stata version 15.1). Forest plots were generated to illustrate the overall effect of knee arthroplasty interventions. *Results*: As a result, it was obtained that there is a significantly higher risk of suffering AKP in the non-resurfacing group, in addition to a significant increase in the risk of undergoing a reoperation in the non-resurfacing group. On the other hand, significant differences were obtained in favor of the resurfacing group in both the clinical and Feller KSS, with functional KSS being inconclusive. After analyzing different variables throughout the literature, it does seem clear that the non-resurfacing group may present a higher risk of reoperation than the resurfacing group. *Conclusions*: For all these reasons, we think that, although it does seem that not replacing the patella can precipitate a reoperation, it is not clear whether this reoperation is a direct consequence of not having replaced the patella. Therefore, in our opinion, the treatment must be individualized for each patient.

## 1. Introduction

Total knee arthroplasty is one of the most common surgeries of our time, being more and more frequent due to the increase in life expectancy and quality of life of patients [1]. There are many possibilities within this type of intervention (one-compartment, two-compartment or three-compartment prostheses). The decision to replace the patella or not has been the subject of debate over the years and remains controversial. On the one hand, the defenders of not replacing the patella or doing it only in selected cases argue that the replacement could increase the rate of patella fracture, rupture of the polyethylene or loosening of the latter, among other complications [2], while those who defend systematic replacement cite less anterior knee pain and a lower revision rate [3,4]. However, it is also indicated that the complications of replacing it may be more severe (fracture or avascular necrosis of the patella) [5,6].

Likewise, the functional results of the different options in terms of patella replacement in knee arthroplasty remain under debate [7,8]. All of this has led to the realization of randomized clinical trials to evaluate the efficacy of the different therapeutic options. Currently, it is still a matter of debate; some surgeons prefer patellaplasty over replacement [9,10], while others prefer selective replacement of the patella based on the state of the cartilage, bone stock or the shape of the patella, radiographic issues or individual characteristics of the patient, such as weight, height or personal preference [9,11].

Over the years, different meta-analyses have been carried out to provide evidence on the subject [8,9,10,11]. However, in recent times, there have not been many new studies in this regard, the last being published in 2019, based on the search criteria. Therefore, it is considered necessary that the latest works form part of a new meta-analysis.

For this reason, the present article includes five new randomized clinical trials of the ten selected in the systematic review, which are not included in previous meta-analyses. We also include some variables that are not very constant throughout the literature, such as the deep infection rate, or radiographic variables such as the Insall ratio or the congruence angle, among others, in addition to the most common variables, such as functional scales, the KSS being the most frequent.

## 2. Materials and Methods

The PRISMA (Preferred Reporting Items for Systematic Reviews and Meta-Analyses) checklist and statement was followed for conducting this study [12,13].

### 2.1. Search Strategy

We searched the literature using PUBMED, SCOPUS, the Cochrane database and BVS from 1 January 2010 to 15 September 2020. The search terms used were “patellar” AND “resurfacing” OR “replacement” AND “non-resurfacing” OR “non-replacement”. The search was performed between 16 September and 20 September.

### 2.2. Inclusion Criteria

English- and Spanish-language literature, prospective randomized control trials (RCTs) performed in humans comparing total knee arthroplasty (TKA) with patellar resurfacing to TKAs without patellar resurfacing. Adults undergoing primary total knee arthroplasty. Data for at least one of the following outcomes: Visual Analog Scale (VAS), Knee Society Score (KSS), WOMAC score, KOOS, Feller score, postoperative outcomes (such as anterior knee pain or deep infection) and radiological outcomes (Insall ratio, congruence angle or patellar tilt).

### 2.3. Data Extraction

Two authors independently extracted data, using standardized data extraction forms, from studies and discrepancies were resolved by discussion and consensus with a third party. Extracted data included publication information, number of knees analyzed, general characteristics of the study (such as mean age, gender, BMI), type of implant, follow-up time and outcomes reported. The number of subjects, mean and SD of continuous outcomes were extracted.

### 2.4. Risk of Bias Assessment

Two of the authors independently assessed the methodological quality of each included study using the Cochrane Collaborations’ tool for assessing risk of bias, and discrepancies were resolved by discussion [14]. This checked for random sequence generation, allocation concealment, blinding of participants and personnel, blinding of outcomes assessors, incomplete outcome data and selective reporting [15].

### 2.5. Meta-Analysis

A meta-analysis was performed using the Stata Software for Statistics and Data Science (Stata version 15.1, College Station, TX, USA: StataCorp LLC) and was limited due to the clinical heterogeneity of the included studies. Forest plots were generated to illustrate the overall effect of knee arthroplasty interventions and funnel plots were produced to assess publication bias.

## 3. Results

**Figure 1:**
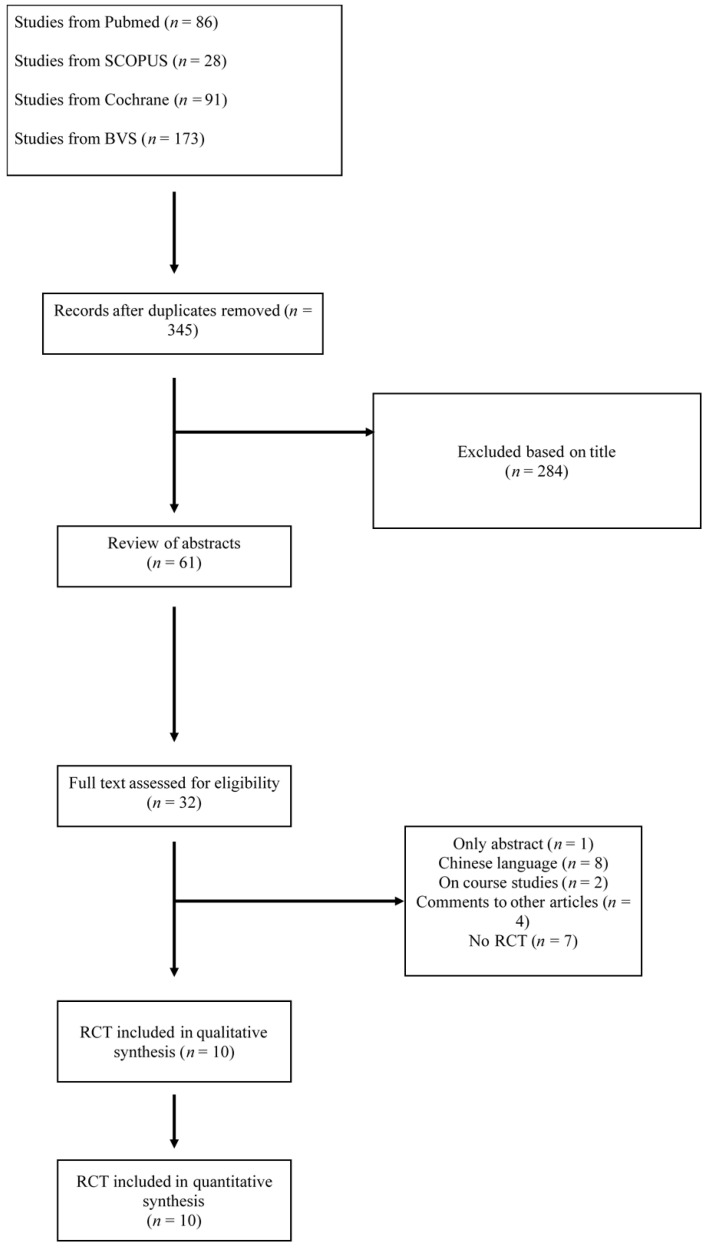
Search flowchart.

The literature search yielded 345 potentially relevant papers (Figure 1). Following screening and the application of eligibility criteria, 61 relevant papers were identified based on title and abstract. These papers were subjected to screening of their full texts. Following application of the eligibility criteria to the full texts, we were able to identify 10 studies for inclusion (Table 1). The assessment of bias for these studies can be seen in Table 2.

It is essential to emphasize that all the exposed variables correspond to the results given after the surgical intervention at its maximum time of follow-up.

- *Range of Motion (ROM)*

**Figure 2:**
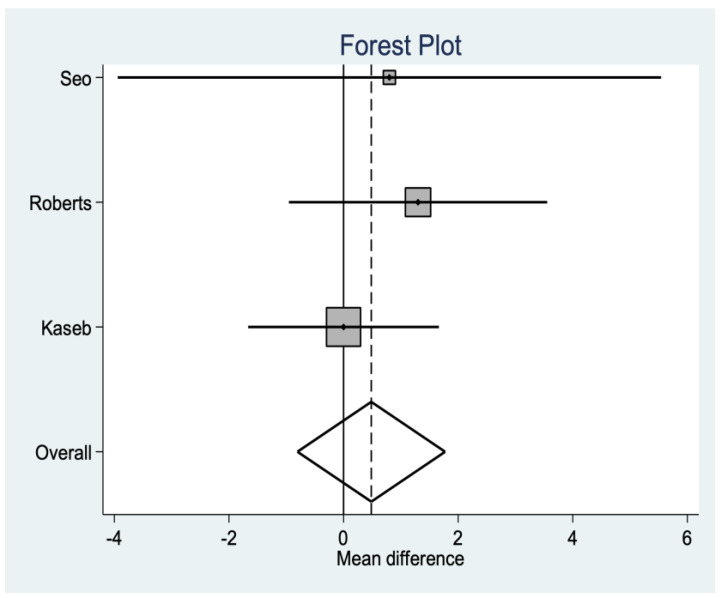
Range of Motion (ROM) data.

There are three studies that provide data on ROM, observing that there are no significant differences between replacing and not replacing the patella, as seen in Figure 2.

- *KSS clinical*

**Figure 3:**
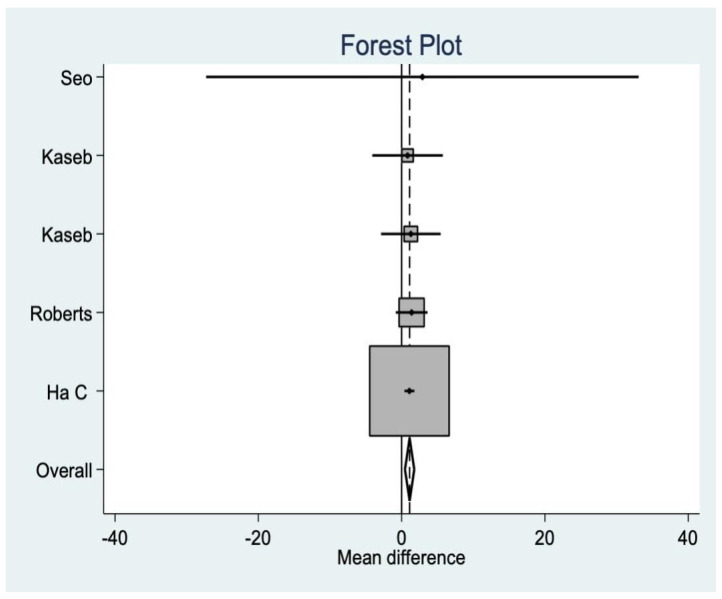
KSS clinical data.

This is the variable that is repeated the most throughout the studies, and statistically significant differences (*p* = 0.0007) are observed in favor of the resurfacing group (Figure 3).

- *KSS functional*

**Figure 4:**
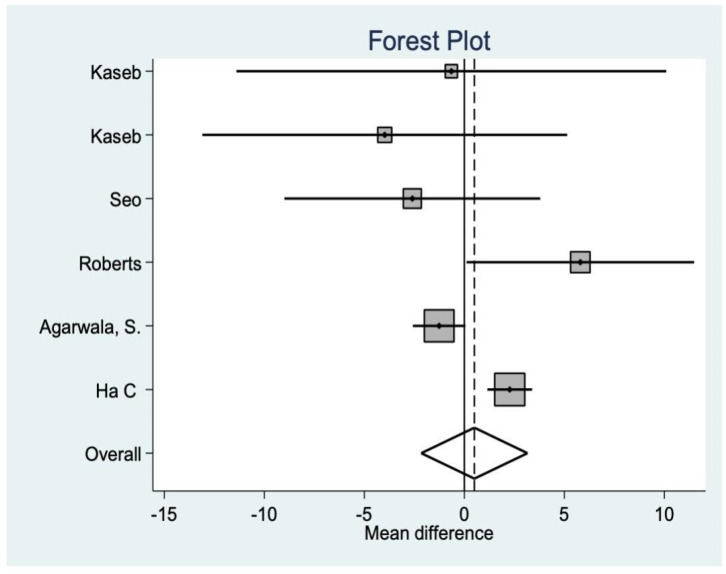
KSS functional data.

This variable is also found quite consistently in the literature, but, in this case, we did not find significant differences (Figure 4 ).

- *Feller Score*

**Figure 5:**
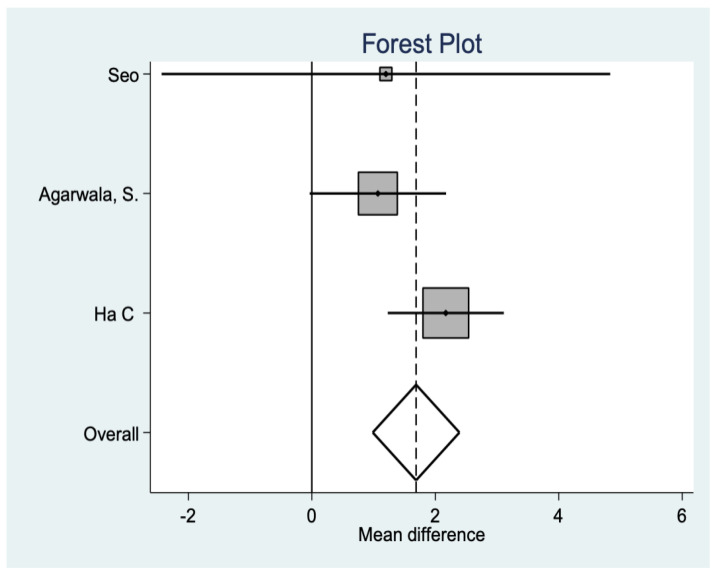
Feller score data.

This is another variable that analyzes functionality after the intervention, finding, in this case, significant differences in favor of the resurfacing group (*p* = 0.001 (Figure 5).

- *Visual Analog Scale (VAS)*

**Figure 6:**
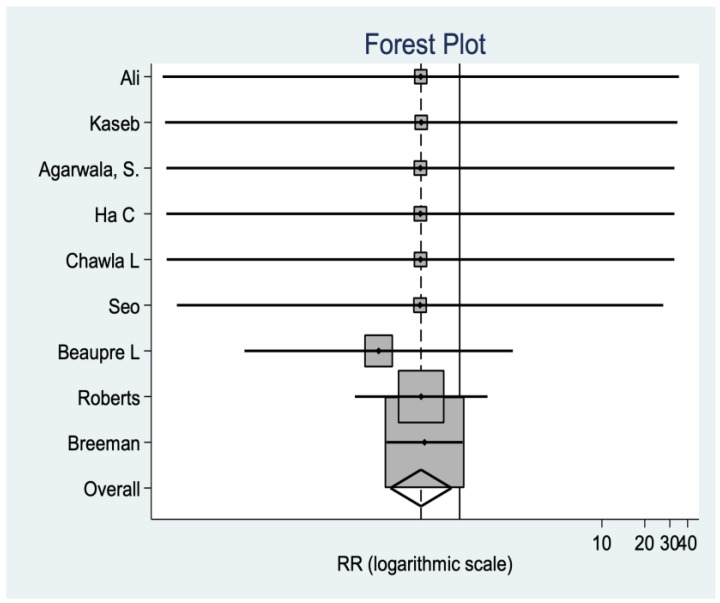
Visual Analog Scale data.

In three studies, the VAS was analyzed for postoperative pain, where no significant differences were found between groups (Figure 6).

- *Review fee*

**Figure 7:**
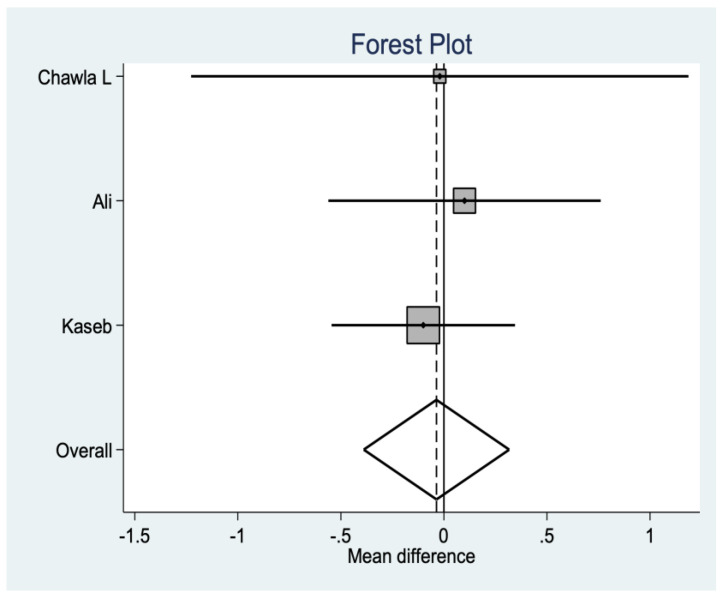
Review fee data.

Data are reported in this regard in nine studies, observing a significant difference in favor of the no-resurfacing group (*p* = 0.0137). In other words, this group has a higher risk of revision than the resurfacing group (Figure 7).

- *Deep infection rate*

Regarding the deep infection rate, a precise statistical analysis cannot be performed due to the few reported cases, since only one case of infection has been reported in the non-resurfacing group [19] and another case in the resurfacing group [18]. The rest of the studies that report data on this question did not find cases of infection in either of the two groups.

- *Anterior Knee Pain (AKP)*

**Figure 8:**
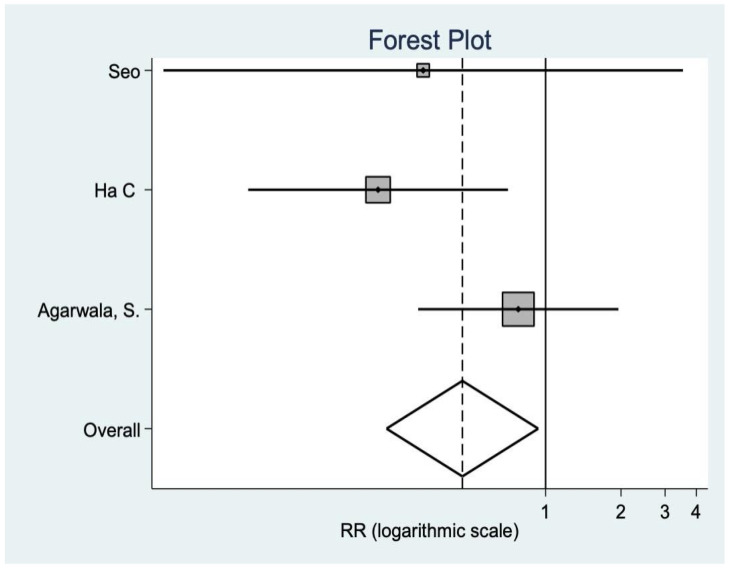
Anterior knee pain data.

There are results in three of the studies, which show statistically positive data in favor of no resurfacing (*p* = 0.012). In other words, in this group, there is a greater risk of suffering from AKP than in the resurfacing group (Figure 8).

- *Radiological variables*

**Figure 9:**
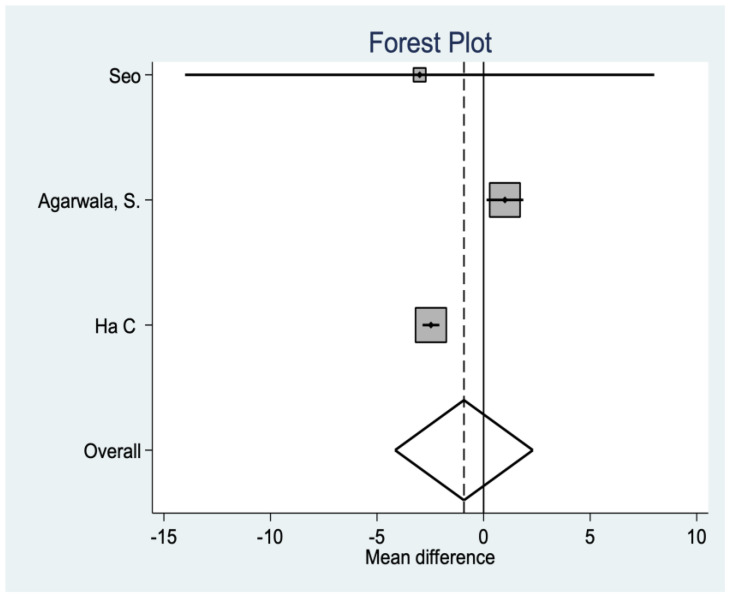
Radiological variable data.

**Figure 10:**
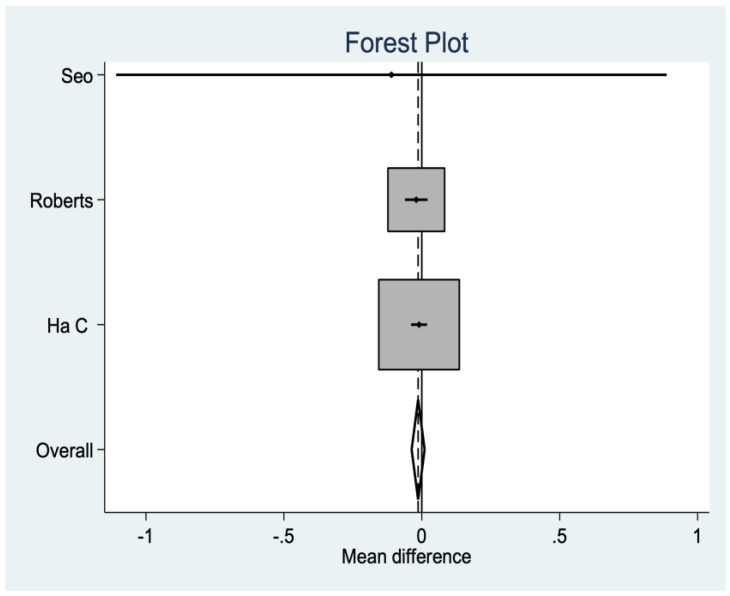
Radiological variable data.

Radiological variables are not very constant throughout the literature. In this case, both the Insall ratio and the patellar tilt are found in three studies that show that there are no statistically significant differences between groups (Figure 9 and Figure 10).

Although the clinical effect of each variable is not analyzed in any of the studies included in the meta-analysis, it seems reasonable to suggest that purely clinical variables such as ROM or functionality scales should have greater weight in daily clinical practice than, for example, the radiological variables studied, which have an application that is more academic than practical. This correlation between the variables is reflected in Table 3. The data obtained are summarized in Table 3.

## 4. Discussion

The question of whether to replace the patella in a total knee replacement has been debated over the years. Several meta-analyses or systematic reviews of the literature have been conducted in an attempt to try to elucidate which is better. The most critical aspects seem to be the reduction of anterior knee pain and better functional outcomes in terms of the option to replace the patella, as opposed to complications such as patella fracture or loosening.

One of the most important variables is the revision rate, in which a greater tendency is observed in the non-resurfacing group, but, as has been postulated throughout the study, it does not seem clear that this higher revision rate could be attributable to patella replacement. In fact, this is the key point of the discussions in favor of one trend or another throughout the entire body of literature.

Regarding anterior knee pain, He et al. [26] reported a higher rate of AKP in the non-resurfacing group but without being statistically significant. Li et al. [27], in studies with more than 5 years of follow-up, did find significant differences, although the data were not consistent in the subgroup analysis. It seems that similar data were maintained throughout the literature [28,29,30], with varying degrees of statistical significance. Meanwhile, others, such as Arirachakaran et al. [31,32], observed that patellar denervation had a lower risk of AKP than patellar replacement, although these results were not statistically significant.

Longo et al. [33,34] more recently did observe significant differences in AKP as it was statistically higher in the non-resurfacing group, while other studies, such as Tang [35] or Teel [36], found no differences between groups.

In our study, we did find a significantly higher risk of AKP in the non-resurfacing group.

As we can see, it seems that, in the literature consulted, there are more studies that consider that anterior knee pain occurs more commonly in the non-resurfacing group than the other way around. Although we do not consider this to be critical in day-to-day clinical practice, the risk–benefit of systematically replacing the patella to avoid AKP, with the possible complications of AKP, should be studied.

The reoperation rate is another of the most studied variables in the literature. He [26] found a higher reoperation rate for patellofemoral problems in the non-resurfacing group. Li et al. [27] observed a higher reoperation rate, both absolute and due to femoropatellar problems, in the non-resurfacing group, with statistically significant results. Fu et al. [28] observed that the absolute risk of reoperation was reduced by 4% in the resurfacing group, meaning that approximately 25 patellae would need to be replaced to prevent one case of reoperation. Similar data were presented by Pavlou et al. [29], Chen et al. [31], and others [32,34,35,36,37,38].

In our case, a significantly higher risk of reintervention was observed in the non-resurfacing group.

On this point, it is true that most of the studies speak in favor of patellar replacement and we do consider in this case that it is something to be considered in clinical practice. We also believe that it should be studied in more depth because, although some studies do distinguish between cases due to the patella and those not due to the patella in their reoperation rates, others do not. The consequences and the magnitude of these consequences should also be considered.

The results of many meta-analyses consulted [26,27,28,29,31,33] showed no differences between groups. However, Pilling et al. [30] did observe differences in favor of the resurfacing group in KSS, and Teel [15] observed differences in both KSS and functional scores. Meanwhile, Aririchakaran [32] obtained worse results in the KSS functional score in the resurfacing group, but this was not significant. Longo et al. [34] did not observe differences in most of the functional tests but did find that the resurfacing group had statistically higher values in the HSS.

Likewise, Tang et al. [35] found no differences in the KSS, although, in the analysis by subgroups, they did observe that, in studies with 1–2-year follow-up, the resurfacing group presented significantly better data, which did not occur when the follow-up was longer than 2 years. In our case, we did find differences in favor of the resurfacing group in both KSS clinical and Feller, with KSS functional being inconclusive.

Here, again, there is a lack of consensus, with most studies finding a lack of differences between groups.

## 5. Conclusions

As stated in the Introduction, there are not many methodologically sound meta-analyses that include modern studies. In fact, this includes five modern clinical trials not found in other meta-analyses.

After analyzing different variables throughout the literature, it does seem clear that the non-resurfacing group may have a higher risk of reintervention than the resurfacing group. In terms of functional variables, we found some disparity. On some occasions, they seem to be in favor of the resurfacing group, as in our study, and others are not; thus, it seems sensible to suggest that these types of variables should not excessively condition the results regarding whether the patella is replaced or not replaced in a total knee prosthesis.

As for the radiological variables and the VAS of pain, no significant differences were observed, so they do not seem to have a great influence on the results either.

As a limitation, we note that the period of inclusion of the studies was limited to 10 years, with the possibility of omitting suitable studies carried out previously, although it is true that some of the meta-analyses consulted included previous studies.

One of the main strengths is the inclusion in the meta-analysis of the most recent clinical trials found on the subject, in addition to the analysis of variables not previously analyzed but which may be of great interest, such as the rate of deep infection, a serious clinical complication for both the patient and the surgeon. Since there are few studies that report this rate as such, which we consider to be of great clinical interest, we believe that our work indicates that it should be included in future studies.

For all these reasons, we believe that, although it seems to have been demonstrated that not replacing the patella may precipitate a reintervention, we do not believe that it is necessary to replace the patella every time, because it is not clear whether this reintervention is a direct consequence of not replacing the patella or not, and doing it systematically may lead to greater consequences.

Therefore, in our opinion, treatment should be individualized for each patient.

## Figures and Tables

**Table 1:** Articles selected and included in the meta-analysis.

First Author	Nº Of Knees Analyzed (Pr/Npr)	Mean Age (Pr/Npr)	Type Of Implant	Mean Follow-Uptime	Outcomes
Breeman S. 2011 (UK) [16]	1715 (818/897)	70/70	At surgeon’s discretion	5 years	OKS, SF-12, EQ-5D, costs, revision, patellofemoral complications
Seo S.S. 2011 (Korea) [17]	277 (168/109)	67.2	E-motion CR Braun	74.6 months	ROM, KSS, HSS, Feller score, AKP, revision, lnsall ratio, congruence angle, patellar tilt.
Beaupre L. 2012 (Canada) [18]	38 (21/17)	(64.9/62)	Smith & Nephew Profix	10 years	ROM, WOMAC, revision, deep infection, RAND-36
Roberts D.W. 2015, (USA) [19]	350 (178/172)	(70.2/71.3)	DePuy Sigma	10.4 years	KSS, ROM, AKP, revision, deep infection, lnsall ratio
Ali A. 2016 (Sweden) [20]	69 (35/39)	68/69	Stryker Triathlon	6 years	VAS, KOOS, revision
Agarwala, S. 2018 (India) [21]	120 (60/60)	(64.17/65.2)	Zimmer NexGen	(18.8/19.2) months	KSS, Feller score, MSMCS, revision, deep infection, AKP, congruence angle, patellar tilt
Kaseb M.H. 2018 (lran) [22]	50 (24/26)	64.8	Zimmer	6 months	ROM, KSS, AKPS, WOMAC, SF-36, VAS, revision, patellofemoral complications, deep infection
Kaseb M.H. 2019 (lran) [23]	73 (29/44)	(68.1/65.75)	Zimmer NexGen	(8.1/9.34) months	KSS, KOOS
Ha C. 2019 (China) [24]	120 (60/60)	65.2	Stryker Scorpio	66.4 months	KSS, Feller score, revision, patellofemoral complications, AKP, lnsall ratio, Patellar tilt
Chawla L. 2019 (India) [25]	100 (50/50)			5 years	KSS, VAS, revision, patellofemoral complications

**Table 2:** Assessment of study bias (red=high bias, yellow=intermediate bias, green=low bias.

	Random Se- Quence Ge Neration (Se Lection Bias)	Allocation Concealment (Selection Bias)	Blinding Of Participants And Person- Nel (Perfor Mance Bias)	Blinding Of Outcomes Assessment (Detection Bias)	Incomplete Outcome Data (Attri- Tion Bias)	Selective Reporting (Reporting Bias)
Breeman 2011 [16]	-	+	?	?	-	-
Seo SS 2011 [17]	-	?	-	-	-	-
Beaupre 2012 [18]	-	-	-	-	-	-
Roberts 2015 [19]	-	-	-	-	-	-
Ali 2016 [20]	?	?	?	-	-	-
Agarwala 2018 [21]	?	+	?	?	-	-
Kaseb 2018 [22]	-	+	-	-	-	-
Kaseb 2019 [23]	?	?	?	?	-	-
Ha 2019 [24]	-	-	-	-	-	-
Chawla 2019 [25]	-	?	?	?	-	-

**Table 3:** Summary of results.

Variable	Outcome
ROM	No differences
KSS clinical	Better resurfacing
KSS functional	No differences
Feller	Better resurfacing
VAS	No differences
Revision	More for non-resurfacing
AKP	More for non-resurfacing
Radiological	No differences

## Data Availability

The study did not report any data.
